# Flavonols do not affect aphid load in green or senescing birch leaves but coincide with a decrease in Photosystem II functionality

**DOI:** 10.1242/bio.060325

**Published:** 2024-07-02

**Authors:** Heta Mattila, Sergey Khorobrykh, Esa Tyystjärvi

**Affiliations:** ^1^ Department of Life Technologies/Molecular Plant Biology, University of Turku, Itäinen Pitkäkatu 4 C 6th floor, 20520 Turku, Finland; ^2^ Centre for Environmental and Marine Studies, University of Aveiro, Campus Universitário de Santiago, 3810-193 Aveiro, Portugal

**Keywords:** *Betula*, Coevolution, *Euceraphis betulae*, Flavonoid, Light stress, Plant-insect interaction

## Abstract

Instead of red anthocyanins, birches synthesise colourless (to human eye), UV-absorbing flavonols during autumn senescence. To test if flavonols protect against insects, and if leaves with high or low amounts of flavonols differ in their photosynthetic functions, aphid-free and aphid-infested green and senescing birch leaves were collected from outdoor-grown trees and analysed. Photosynthetic parameters were greatly affected by the leaf chlorophyll content (i.e. the phase of senescence). Photochemical quenching and the amount of functional Photosystem I decreased linearly with chlorophyll content, while F_V_/F_M_ (Photosystem II functionality) decreased strongly only at the end of senescence. Non-photochemical quenching of excitation energy (NPQ) increased towards the end of senescence. However, no significant differences in the total flavonol amounts, nor in individual flavonol species, were found between aphid-free and aphid-infested leaves, suggesting that flavonols play no role in defence against aphid herbivory. Interestingly, both green and senescing leaves with a high flavonol content showed low F_V_/F_M_ values. High flavonol content slowed down PSII photoinhibition and improved recovery, but only in green leaves. Previously, we proposed that anthocyanins provide an additional sink for photosynthates at the nitrogen resorption phase during autumn senescence, and the present data may suggest that flavonol synthesis plays a similar role.

## Teaser

**Summary:** We measured colourless, UV-absorbing flavonols and photosynthetic parameters in aphid-free and aphid-infested green and senescing birch leaves. Flavonols did not affect aphid amounts but correlated negatively with Photosystem II activity.

## INTRODUCTION

The bright colours of autumn leaves have been suggested to function as a (warning) signal for insect herbivores, either signalling for a high investment in defence (Hamilton and Brown, 2001) or for poor food quality (Archetti, 2000). These hypotheses, especially in their original formulations, have also received criticism, e.g. for treating both red and yellow leaves as ‘bright’ or for not taking into account insects' vision and actual colour preferences. During senescence, chlorophylls are often degraded faster than carotenoids, which unmasks the yellow colours of carotenoids, while red colours are due to synthesis of anthocyanins. Thus, red colours require an investment from the senescing plant while yellow colours may be regarded as a side effect of chlorophyll degradation. It should also be noted that the eyes of most insects, e.g. those of the green peach aphid (*Myzus persicae*), have photoreceptors for UV-radiation and for blue and green light but probably not for red light (Kirchner et al., 2005; for a review, see Döring and Chittka, 2007). Consequently, to insects, yellow leaves may indeed appear bright while ‘bright’ red leaves may look rather dull. In addition to colour, insects may recognise senescing leaves via olfactory signals (Glinwood and Pettersson, 2000). Besides signalling low-quality food, red (autumn) colours may camouflage leaves, undermine insect camouflage, attract the enemies of insect herbivores or indicate that the leaf will die soon (see Wilkinson et al., 2002; Yamazaki, 2008; White, 2009; Lev-Yadun and Holopainen, 2009; Archetti et al., 2009a; Hughes et al., 2021; Pena-Novas and Archetti, 2022). Importantly, the hypotheses explaining autumn colours by plant-insect interactions often do not exclude other roles, such as protection against high light, for the anthocyanin synthesis (see, e.g. Agati et al., 2021; Hughes et al., 2022). For an in-depth discussion of leaf colours and insect herbivory, see the recent review by Lev-Yadun (2022).

Aphids are phloem-sucking insect herbivores that colonise new hosts during late summer or early autumn, and mate and lay eggs that overwinter on the tree (e.g. Heie, 1982; Furuta, 1986). Aphid infestation can severely reduce tree growth (Dixon, 1971; Zvereva et al., 2010; Sinkkonen et al., 2012). Due to increased loading of amino acids into the phloem, yellow (i.e. senescing) leaves are a rich food source for sap-sucking insects (Holopainen and Peltonen, 2002; Farnier and Steinbauer, 2016). It has also been suggested that, compared to yellow leaves, green leaves produce more volatile compounds that attract aphid predators (Holopainen, 2008; Holopainen et al., 2010). Indeed, aphids are often more attracted towards yellow leaves than towards green leaves (Kennedy et al., 1961; Furuta, 1986; Ramírez et al., 2008; Döring et al., 2009; Sinkkonen et al., 2012; Farnier et al., 2014; Farnier and Steinbauer, 2016), although some aphid species prefer green leaves in the autumn (Archetti and Leather, 2005).

Between yellow and red senescing leaves, aphids seem to avoid red leaves (Furuta, 1986; Ramírez et al., 2008; Archetti, 2009). Furthermore, anthocyanins have been observed to accumulate after an infestation of sap-sucking insects (Costa-Arbulú et al., 2001; Sun et al., 2016; Steinbauer et al., 2018). Red leaves often contain high amounts of potential defence compounds (e.g. Green et al., 2015). Indeed, a chrysanthemum (*Chrysanthemum morifolium*) mutant with decreased flavonoid content was found to be susceptible to the chrysanthemum aphid (*Macrosiphoniella sanborni*) (Wang et al., 2024). However, leaf anthocyanin content did not affect the fecundity or survival of the yellow sugarcane aphid (*Sipha flava*) (Costa-Arbulú et al., 2001) or the growth of the peach aphid (*Tuberocephalus momonis*). Anthocyanins instead protected the peach aphid from UV-B radiation (Zhou et al., 2020). Furthermore, in soybean (*Glycine max*), grazing insects caused a bigger increase in anthocyanin accumulation than an aphid infestation (O'Neill et al., 2010).

Silver birch (*Betula pendula*) does not turn red during autumn senescence, but accumulation of flavonols coincides with chlorophyll degradation (Mattila et al., 2018). Another mainly yellow-senescing species, English oak (*Quercus robur*), behaves similarly (Brelsford et al., 2022). An increase in flavonol content in the autumn has been observed also in some red-senescing species, such as in Norway maple (*Acer platanoides*) (Mattila et al., 2018; Brelsford et al., 2022), but not in bird cherry (*Prunus padus*), nor in common grape wine (*Vitis vinifera*) (Mattila et al., 2018; Sitko et al., 2019). Flavonols are invisible to human eye but absorb UV-radiation. Indeed, flavonol synthesis is often induced by UV-radiation, also in silver birch (Morales et al., 2010). In some deciduous species, the autumnal flavonol accumulation decreases if the amount of UV-radiation is experimentally reduced (Brelsford et al., 2022). However, little is known as to why flavonols increase during the autumn.

Although flavonols do not absorb visible light, they are not transparent from an insect (aphid) viewpoint, as insects can usually see UV-radiation. Furthermore, flavonols might deter aphids by smell or taste as aphids use also olfactory and chemical cues to find and select host leaves (for reviews, see Döring, 2014; Nalam et al., 2019). Indeed, it has been suggested that the presence of certain flavonol species in plant leaves plays a role in aphids' recognition of host leaves (Takemura et al., 2002). Accordingly, exogenous application of the flavonols quercetin and rutin enhanced or delayed probing, depending on the aphid species (Stec et al., 2021). The flavanone naringenin and quercetin have also been shown to be harmful to aphids (Goławska et al., 2014); however, the applied concentrations may have been too high to indicate biological significance. In cassava (*Manihot esculenta*), the amount of flavonols in phloem sap was observed to increase after infestation by the sap-sucking cassava mealybug (*Phenacoccus manihoti*) (Calatayud et al., 1994). In broccoli (*Brassica oleracea*), in contrast, an aphid infestation did not change flavonol levels (Khan et al., 2011). In tea plants (*Camellia sinensis*), infestation by a moth (*Ectropis grisescens*) lead to increased glucosylation of quercetin, and only quercetin glucoside (not the free quercetin) inhibited the growth of the moth's larvae (Jing et al., 2024). Unfortunately, insect fitness has only rarely been studied with senescing leaves.

It has not been thoroughly investigated, if variation in chemical compositions within yellow (i.e. not between yellow and red) senescing leaves affects insect herbivory. For example, it is not well understood how UV-absorbing compounds, such as flavonols, affect the insect's survival, reproductive success or selection of host leaves (see Sinkkonen, 2009; Archetti et al., 2009b). Here, we compared senescing and green birch leaves that contained different amounts of flavonols, in an attempt to test whether aphids prefer certain yellow leaves over others. In addition, several photosynthetic parameters were measured to see how photosynthesis is affected by senescence, aphid load and flavonol content.

## RESULTS

### Aphids were found on both green and senescing birch leaves

Aphid-free and aphid-infested birch leaves were picked during the autumns 2021 and 2022 from trees growing in small city parks. For quantification purposes, the collected leaves were classified as non-senescing (green), or senescing (yellow) based on their chlorophyll content (Table 1). Aphids were usually easier to find on senescing leaves than on green leaves, which was reflected in the higher proportion of aphid-free samples among green leaves than among senescing leaves (Table 1). In the present data, nymphs were more abundant than winged adults but about half of the aphid-infested leaves also contained winged adults, both in the case of green and senescing leaves (Tables 1,2). Most commonly, 5-6 nymphs and a winged adult resided on an aphid-infested leaf, but a large variation was observed; up to 68 nymphs were found on a single leaf (Table 2). In the present data, the average number of aphids on a single aphid-infested leaf was fairly similar between senescing and green leaves (Table 2). To analyse the data, statistical models were built. As the number of aphids on a leaf was over-dispersed (dispersion 15.3, *P*=2.2×10^−6^; Table S1), a model assuming a negative binomial distribution of the response variable, instead of Poisson distribution, was chosen. In addition, a Poisson distributed model for the mere presence of aphids on a leaf was built. As expected, based on Table 2, neither of the models showed that leaf chlorophyll content would have a significant effect on aphid infestation (Table 3; Tables S2 and S3).

**Table 1.**
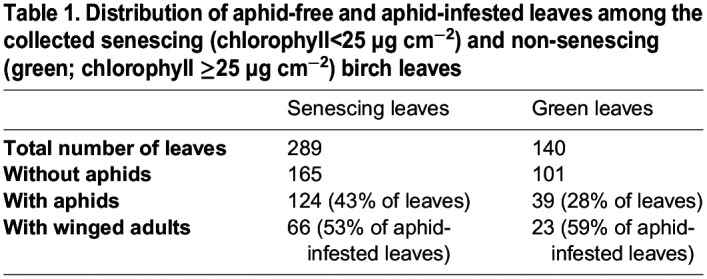
**Distribution of aphid-free and aphid-infested leaves among the collected senescing (chlorophyll<25** **µg** **cm^−2^) and non-senescing (green; chlorophyll ≥25** **µg** **cm^−2^) birch leaves**

**Table 2.**
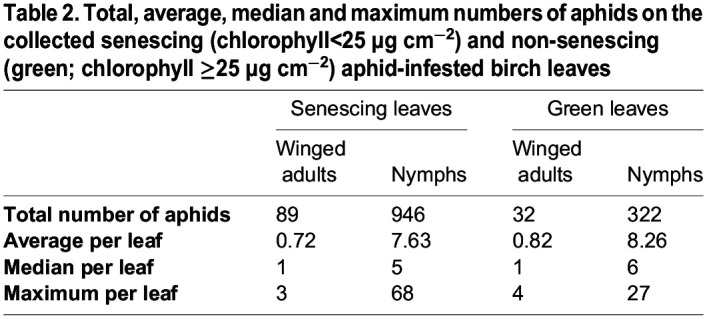
**Total, average, median and maximum numbers of aphids on the collected senescing (chlorophyll<25** **µg** **cm^−2^) and non-senescing (green; chlorophyll ≥25** **µg** **cm^−2^) aphid-infested birch leaves**

**Table 3.**
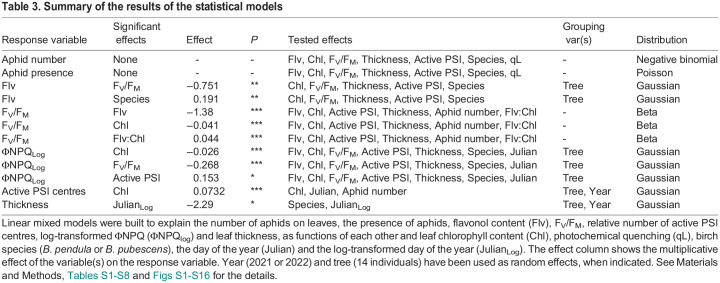
Summary of the results of the statistical models

### Photosynthetic parameters were greatly affected by leaf senescence

**Fig. 1.**
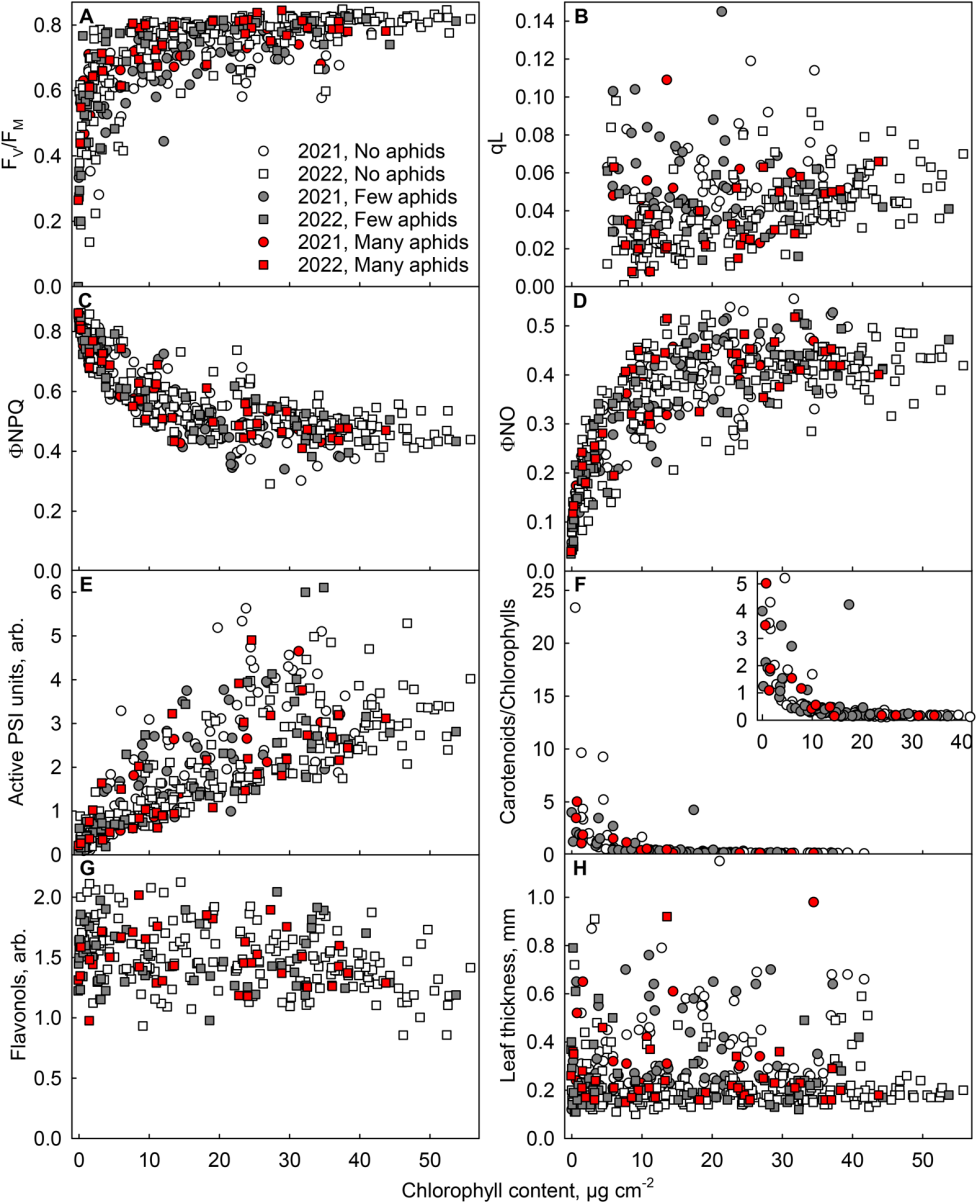
**Physiological parameters measured from birch leaves with different chlorophyll contents and aphid loads.** Open symbols, no aphids detected on the leaf; grey symbols, few, less than 10 aphids, red symbols, many, 10 or more aphids. Leaves were collected during the autumns of 2021 (circles) or 2022 (squares). F_V_/F_M_ was measured after 30 min in the dark (A). Photochemical quenching (qL; B), yields of NPQ (C) and NO (D) and the amount of active PSI centres (arbitrary units) (E) were measured under light [photosynthetic photon flux density (PPFD) 1000 µmol m^−2^ s^−1^]. Measurements of qL from leaves with low chlorophyll content (<5 µg cm^−2^) have been removed. The ratio of carotenoids to chlorophylls (F) was measured spectrophotometrically after pigment extraction in methanol. The inset in F shows the same data with a different Y-axis scale, to make the data points more visible. Leaf total flavonol contents (G), measured with an optical method (Dualex). (H) Leaf thickness. In all figures, except in F, chlorophyll contents were measured with an optical method (MultispeQ) and converted into µg cm^−2^ with an empirical calibration curve. Each symbol represents an individual measurement, from an individual leaf (*n*=429 for A, C-E and H; *n*=327 for B; *n*=141 for F; n=287 for G), collected from 14 trees.

Next, several photosynthetic and other physiological parameters were measured from the collected leaves. Values of the fluorescence parameter F_V_/F_M_ decreased with decreasing chlorophyll content, though initially very slowly (Fig. 1A). A linear model assuming beta distribution for the response variable F_V_/F_M_, analysing the effects of flavonol content, chlorophyll content, relative amount of active PSI centres and leaf thickness, confirmed a significant dependence of F_V_/F_M_ on leaf chlorophyll content (Table 3; Table S4). Also, photochemical quenching (qL) decreased in senescing leaves; however, all measured values were close to zero due to the high light intensity and a short acclimation period used for the measurement. In addition, yield of non-regulated dissipation of light energy (ΦNO), the amount of functional PSI centres and leaf thickness decreased in senescing leaves, whereas yield of regulated energy dissipation (ΦNPQ) and the carotenoids to chlorophyll ratio increased (Fig. 1; for statistics of ΦNPQ and active PSI centres, see Table 3 and Tables S5-S6). Leaf chlorophyll content had a significant positive effect (coefficient 0.073, *P*<2.2×10^−16^) on the number of active PSI centres and a significant negative effect on ΦNPQ (coefficient −0.002, *P*<2.2×10^−16^). In addition, the model for ΦNPQ showed a negative effect of F_V_/F_M_ (coefficient −0.307, *P*<2.2×10^−16^). To test if leaf thickness is a function of the other measured variables, a linear mixed model of the dependence of leaf thickness on the birch species (*B. pendula* or *B. pubescens*) and day of the year was constructed, with the year and tree individual as random effects (Table 3; Table S7). The analysis confirmed that leaf thickness decreased as the autumn progressed (coefficient −2.29, *P*=0.017).

### Flavonol contents did not differ between aphid-free and aphid-infested leaves

Leaf aphid load, on the other hand, clearly had a much smaller effect on the above-mentioned photosynthetic parameters (Fig. 1). The statistical models testing flavonol and chlorophyll content, photosynthetic parameters, leaf thickness, birch species (*B. pendula* or *B. pubescens*) or day of the year as potential effectors for the number of aphids on a leaf (Table 3; Table S2) or for the presence of aphids on a leaf (Table 3; Table S3) did not reveal any significant relationships between leaf aphid load and flavonol content, nor between aphid infestation and the other measured parameters.

**Fig. 2.**
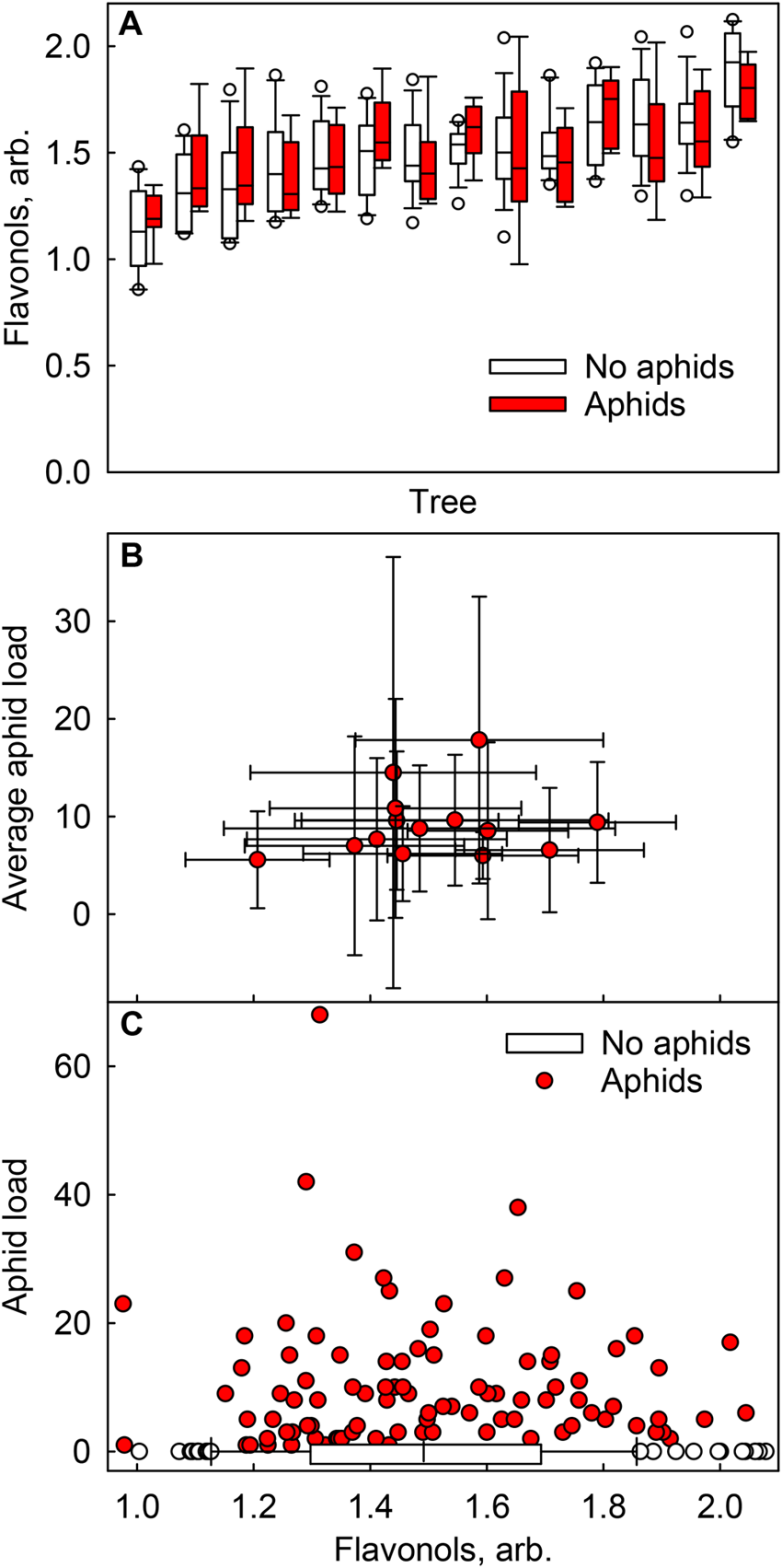
**Flavonol contents and aphid loads of birch leaves collected during the autumn of 2022.** (A) Flavonol contents of aphid-free (open boxes) and aphid-infested (red boxes) leaves in 14 individual trees. (B) Average numbers of aphids and flavonol contents of aphid-infested leaves in 14 individual trees. Error bars show SD, calculated from 12-18 (aphid-free) or 5-9 (aphid-infested) individual leaf measurements. (C) The number of aphids and the flavonol contents of aphid-free (box plot with open circles) and aphid-infested (red circles, individual measurements of individual leaves from 14 trees) leaves. The boxes in A and C show median, 25th and 75th percentiles, error bars show 10th and 90th percentiles and the circles show outliers, calculated based on 12-18 (aphid-free) or 5-9 (aphid-infested) (A) or 139 (C) measurements from individual leaves collected from 14 trees. Flavonols were measured with an optical method (Dualex).

To make sure that differences between trees did not mask any within-tree relationships, average flavonol contents were calculated for each tree, separately for aphid-free and aphid-infested leaves, but no general correlation was found between these two factors (Fig. 2A,B). Furthermore, no correlation could be seen between the number of aphids and the flavonol content of the leaf (Fig. 2C), in line with the statistical modelling (Table 3).

**Fig. 3.**
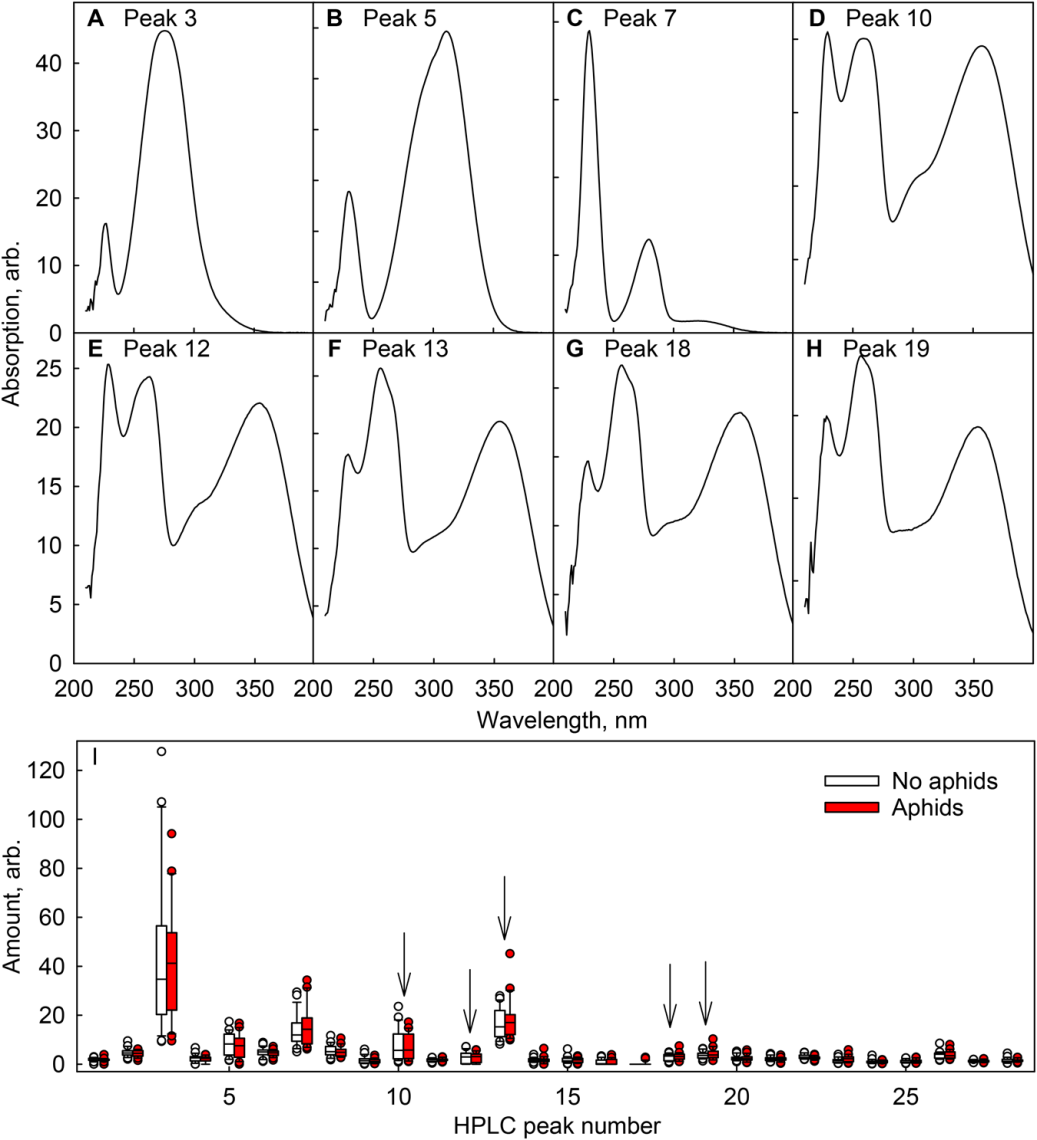
**Flavonoids in birch leaves collected during the autumn of 2021.** Pigments were extracted in methanol and quantified with an HPLC. (A-H) Examples of the spectra of the detected pigments; the spectra of the biggest peaks (3, 5 and 7) as well as those of the probable flavonols (10, 12, 13, 18 and 19; identified based on their absorption spectra) are shown. (I) Quantification of the HPLC peaks (individual pigments; arbitrary units), normalised to leaf dry weight, from aphid-free (open bars) and aphid-infested (red bars) leaves. The boxes in I show median, 25th and 75th percentiles, error bars show 10th and 90th percentiles and the circles show outliers, calculated based on 22-23 measurements from individual leaves, collected from four trees. Arrows indicate the most probable flavonols.

Even if the total flavonol content did not affect leaf aphid loads, certain flavonol species could be enriched or depleted in aphid-infested leaves. Thus, leaf pigments were extracted in methanol and analysed with an HPLC from a set of leaves (Fig. 3). However, the pigment profiles (including the most probable flavonol species) of aphid-free and aphid-infested leaves were very similar (Fig. 3).

### Leaves with high flavonol content showed low F_V_/F_M_ values

**Fig. 4.**
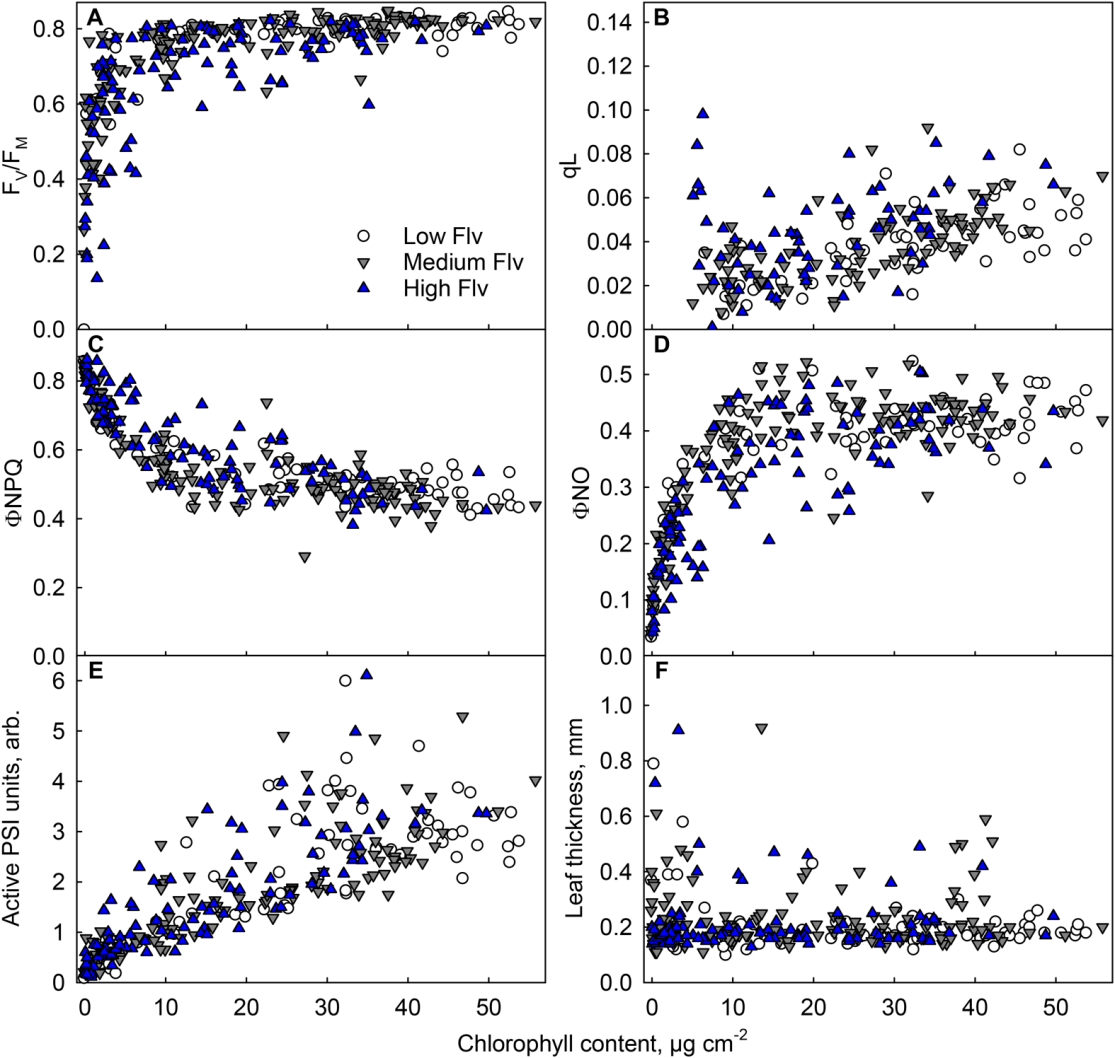
**Physiological parameters of birch leaves containing different amounts of chlorophyll and flavonols.** Open circles, low (<1.3) flavonol (Flv) content; grey downward triangles, medium flavonol content; blue upward triangles, high (≥1.6) flavonol content. Leaves were collected during the autumn of 2022. F_V_/F_M_ was measured after 30 min in the dark (A), photochemical quenching (qL; B), the yields of NPQ (C) and NO (D) and the amount of active PSI centres (arbitrary units) (E) were measured in the light (PPFD 1000 µmol m^−2^ s^−1^). (F) Leaf thickness. Flavonols (Dualex) and chlorophyll contents (MultispeQ) were measured with optical methods; chlorophyll contents were converted to µg cm^−2^ with an empirical calibration curve. Measurements of qL from leaves with very low chlorophyll content (<5 µg cm^−2^) have been removed. Each symbol represents an individual measurement, from an individual leaf (*n*=287, except *n*=212 for B), collected from 14 trees.

Next, effects of the measured physiological parameters on leaf flavonol contents were studied; for a visualisation, leaves were first grouped on the basis of their flavonol contents (Fig. 4). A linear mixed model was built to study the dependence of flavonol content on chlorophyll content, F_V_/F_M_, leaf thickness, amount of active PSI centres and the birch species (*B. pendula* or *B. pubescens*). The tree (14 individuals) was used as a random effect. As expected, based on the descriptive statistics (Fig. 4A), high flavonol content was associated with low F_V_/F_M_ (coefficient −0.292, *P*=0.0068; Table 3; Table S8). Also, the previous model on F_V_/F_M_ (Table 3; Table S4) showed the negative effect of flavonols on the F_V_/F_M_ value (coefficient −1.38, *P*=2.14×10^−15^); the interaction of flavonol content and chlorophyll content also had a negative effect on F_V_/F_M_ (coefficient 0.044, *P*=4.37×10^−9^). In addition, trees varied in their average flavonol contents (as seen in Fig. 2A); the model showed that *B. pendula* had significantly more (coefficient 0.191, *P*=0.0058) flavonols than *B. pubescens*. No further significant effects were found.

### High flavonol content decreased PSII photoinhibition only in green leaves

**Fig. 5.**
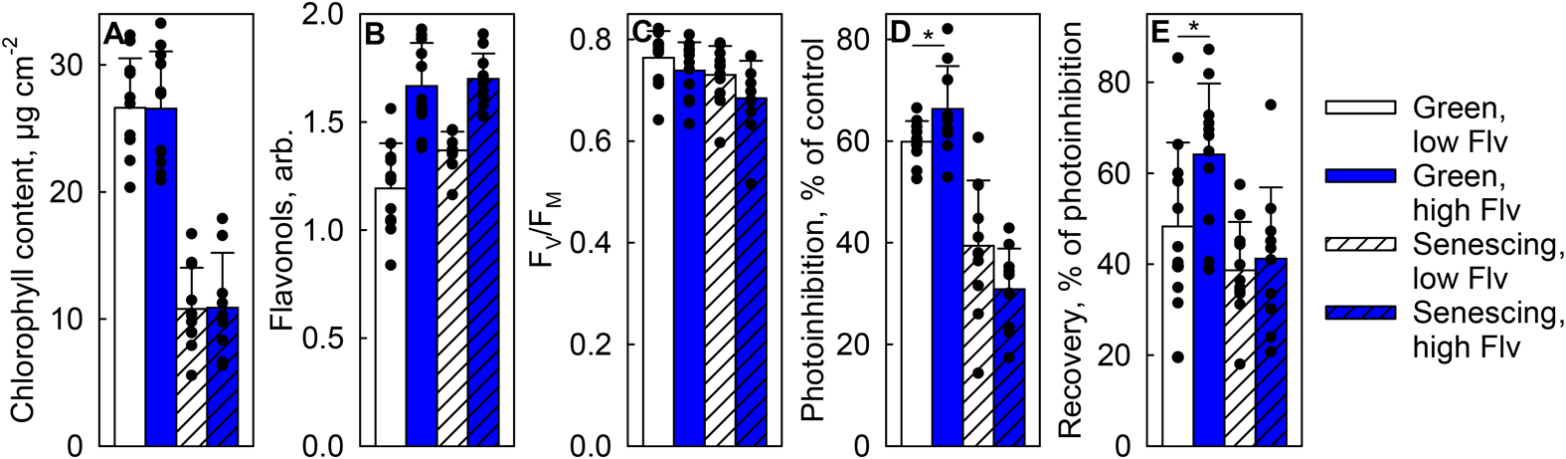
**Effects of flavonols on PSII photoinhibition in birch leaves.** Green (unhatched bars) and senescing (hatched bars) with low (open bars) or high (blue bars) flavonol contents were collected during the autumn of 2022. Chlorophyll (A) and flavonol (Flv; B) contents and control F_V_/F_M_ values (C). Leaves were illuminated for 1.5 h with high light (PPFD 2000 µmol m^−2^ s^−1^) after which PSII photoinhibition was quantified as the decline in the F_V_/F_M_ values (D). Leaves were let to recover for 2 h at low light (PPFD 12 µmol m^−2^ s^−1^) after which the proportion of the photoinhibition that was recovered was quantified (E). F_V_/F_M_ was always measured after 30 min in the dark. Flavonols and chlorophyll contents were measured with an optical method (Dualex); chlorophyll contents were converted to µg cm^−2^ with an empirical calibration curve, as described in Mattila et al. (2018). Bars show averages from 10-11 measurements from individual leaves (shown as circles), collected from four trees and error bars show standard deviation. Statistically significant differences (calculated only for D and E within green and senescing leaves with Mann–Whitney *U*-tests) between the indicated bars have been highlighted with asterisks.

To understand the origin of the low F_V_/F_M_ values in leaves with high flavonol contents (Table 3), green and senescing leaves with either low or high flavonol contents were selected, by picking the leaves with the highest and the lowest flavonol content among the green or senescing leaves of each of the four trees used (Fig. 5A,B), and subjected to a high light treatment and to subsequent low light recovery period. In this case, the control PSII activity (prior any high light treatment), probed by the F_V_/F_M_ parameter, did not statistically significantly differ between leaves of different flavonol contents (Fig. 5C), although a similar trend as before (Table 3; Fig. 4A) was observed. As expected, based on the literature, the F_V_/F_M_ values decreased faster (during the high light treatment) in senescing leaves than in green leaves (Fig. 5D), and also the recovery was slightly less efficient in senescing leaves (Fig. 5E). More interestingly, green leaves of the high flavonol content group experienced less (*P*=0.049) photoinhibition and recovered better (*P*=0.036) than green leaves with low flavonol contents (Fig. 5D,E). However, no statistically significant differences were found between senescing leaves with different flavonol contents.

## DISCUSSION

### The autumnal increase of flavonols in senescing leaves is probably not related to aphids

Improved defence against herbivorous insects is one of the hypothetical fitness advantages suggested to explain flavonoid synthesis in senescing tree leaves (Archetti, 2000; Hamilton and Brown, 2001; Wilkinson et al., 2002; Yamazaki, 2008; White, 2009; Lev-Yadun and Holopainen, 2009; Archetti et al., 2009a; Lev-Yadun, 2022). The defence hypothesis is usually based on the red colours of anthocyanin-containing leaves. In the present study, we tested the defence hypothesis with birch, a species that produces flavonols during autumn senescence. Thus, aphid-free and aphid-infested leaves were collected from senescing trees. Aphid species were not characterised but *Euceraphis betulae* Koch has been shown to be the most common birch aphid in Finland (Holopainen et al., 2009; see also Fig. S17). *E. betulae* prefers yellow (senescing) leaves over green leaves (Holopainen et al., 2009; Sinkkonen et al., 2012). Accordingly, we collected more aphid-infested leaves among senescing leaves than among green leaves Tables 1 and 2). The number of aphids on an aphid-infested leaf, on the other hand, did not statistically differ between senescing and green leaves (Table 3), suggesting that the aphids survive equally well on both green and yellow leaves.

However, we did not find any connection between leaf aphid load and total flavonol content, nor between aphid load and any particular flavonol species (Figs 1-3; Table 3). The data suggest that in birch, flavonols are not synthesised as aphid deterrents. It could be argued that the differences in flavonol contents between the measured leaves (ranging from ∼1-2; arbitrary units) may not have been large enough to cause differences in aphid behaviour. However, the optical method used does not respond linearly to leaf flavonol content (Mattila et al., 2018), and thus, the actual differences in the flavonol amounts, in the present data, may have been bigger than the optical measurements suggest. Furthermore, a relatively small difference (∼30%) in total flavonoids can cause a clear difference in the susceptibility to aphids (Wang et al., 2024). On the other hand, *E. betulae*, most probably the most common aphid in the present data set and a specialist aphid of silver birch, may have evolved to deal with the defences of birch. Indeed, in sorghum (*Sorghum bicolor*), an infestation of a generalist aphid (*Schizaphis graminum*) caused a higher induction of flavonoid synthesis than an infestation of a specialist aphid (*Melanaphis sacchari*) (Puri et al., 2023). Furthermore, this flavonoid accumulation reduced the reproductive success of the specialist aphid, while previous infestation with the specialist aphid (no flavonoid accumulation) had no impact on the generalist aphid (Puri et al., 2023). Besides aphids, flavonols might be used to deter other insects in the autumn, as flavonols negatively affect grazing herbivore insects, such as the larva of the gypsy moth *Lymantria dispar* and the butterfly *Pieris brassicae* (e.g. Onkokesung et al., 2014; Martemyanov et al., 2015).

### Could flavonols protect senescing leaves from light, or function as energy escape valves?

F_V_/F_M_ values (reflecting PSII functionality) were lower in leaves with high flavonol content (Figs 4,5; Table 3). Thus, flavonol accumulation may be a stress response, both in green and senescing birch leaves. Indeed, flavonol species are known to be induced under many stress conditions, e.g. in response to cold or drought, also in deciduous tree species (Stark et al., 2015; Popović et al., 2016). Could the autumnal increase in flavonols protect senescing leaves, which seem more vulnerable to the high-light-induced damage than green leaves (Fig. 5; Mattila et al., 2021)? In the present study, F_V_/F_M_ values were low in leaves with high flavonol content, indicating that the amounts of flavonols present in birch leaves were not sufficient to (fully) prevent the decrease in F_V_/F_M_. It can be hypothesised that the low F_V_/F_M_ values might function as a signal to induce flavonol synthesis, in order to mitigate the stress. Similarly as flavonols, anthocyanin accumulation can coincide with low F_V_/F_M_ values, in both green and senescing leaves (Kytridis et al., 2008; Nikiforou et al., 2011; Mattila and Tyystjärvi, 2023). Previously, we proposed that the same (stress) conditions cause both low F_V_/F_M_ values and anthocyanin accumulation in senescing maple leaves, as a causal relationship between these factors was not found (Mattila and Tyystjärvi, 2023).

To more directly assess the photoprotective capability of flavonols, high light treatments were performed. Indeed, green leaves with high flavonol contents were photoinhibited to a lesser degree than green leaves with low flavonol content (Fig. 5). Because these leaves also recovered better (Fig. 5E), and as it is known that the repair reactions are sensitive to reactive oxygen species (Nishiyama et al., 2001; Toriu et al., 2023), flavonol accumulation may have protected the PSII repair by preventing accumulation of reactive oxygen species. Indeed, flavonols are able to quench and scavenge reactive oxygen species. Agati et al. (2007) have presented experimental data suggesting that chloroplast-localised flavonols (di-hydroxy B-ring substituted quercetin and/or luteolin) detoxify singlet oxygen in green leaves of olive trees (*Phillyrea latifolia*). Scavenging of hydrogen peroxide by flavonols in guard cells has been shown to affect stomatal opening (An et al., 2016; Watkins et al., 2017). On the contrast, previously we observed no effect of flavonol content on singlet oxygen production in senescing or green silver birch leaves (Mattila et al., 2021). Furthermore, no protection by high flavonol content was observed in senescing leaves here (Fig. 5). However, the association of high flavonol contents with low F_V_/F_M_ values was observed in both green and senescing leaves (Table 3; Fig. 4), indicating that the feature is not specific to senescing leaves.

Previously, we suggested that anthocyanin synthesis functions as an electron sink, keeping photosynthesis going on under conditions where nutrient translocation requires energy in the forms of ATP and NADPH, but carbon backbones are no longer actively used for biosynthesis (Mattila and Tyystjärvi, 2023). Other studies have also suggested that flavonoid synthesis functions as an energy escape valve (Lo Piccolo et al., 2008; Hernandez and Van Breusegem, 2010; Soubeyrand et al., 2018; Kitao et al., 2024). Neither flavonols nor anthocyanins contain nitrogen and are thus relatively ‘cheap’ for (senescing) leaves. More research is needed to understand if flavonol synthesis can serve this function in both green and senescing leaves.

### Aphid-infested leaves showed few stress symptoms

Photosynthetic parameters did not differ much between aphid-free and aphid-infested leaves (Fig. 1; Table 3). Previous studies have observed low F_V_/F_M_ values in combination with decreased chlorophyll content in aphid-infested leaves (Burd and Elliott, 1996; Kmieć et al., 2018). In such cases, however, the low F_V_/F_M_ values could also have been a consequence of the low chlorophyll content. Previous research on the effects of aphid infestation on carbon fixation, on the other hand, show variable results. For example, a negative effect was found in cotton (*Aphis gossypii*; Heimoana et al., 2023), no effect in sugar beet (*Beta vulgaris*; Hurej and Van Der Werf, 1993) and a positive effect in apple tree (*Malus domestica*; Pincebourde and Ngao, 2021). The increase in photosynthesis in aphid-infested leaves has been suggested to be a compensation mechanism of the plant; by extracting sap and consuming fixed carbon, aphids create an additional carbon sink to which the plant responds by increasing carbon fixation (Larson and Whitham, 1991; Retuerto et al., 2004). While Pincebourde and Ngao (2021) measured increased carbon fixation in the aphid-infested apple tree leaves, they also reported that the growth of the infected seedlings was compromised. At least leaf age, the number of aphids on a leaf (Pincebourde and Ngao, 2021; Heimoana et al., 2023) and the susceptibility of a species/variety (Burd and Elliott, 1996) may explain the variability in observed responses on aphid infestation. Therefore, the relatively low (average) aphid load on the studied leaves (Tables 1,2) and the fact that the aphids probably were birch specialists (i.e. birches are expected to have evolved ways to tolerate these aphids) may explain the lack of obvious effects in the present study.

### Concluding remarks

We did not find evidence supporting the hypothesis that the autumnal flavonol synthesis in birch would be related to defence against aphids. Instead, we speculate that flavonol synthesis may function as a carbon sink for senescing leaves under stress conditions. However, other possible functions, such as protection against excess light, cannot be excluded. Actually, the fact that flavonols absorb UV-radiation (and other flavonoids absorb also at the visible range) may make these compounds more attractive (than other secondary metabolites, including volatile compounds) because, besides the hypothesised sink function, they could additionally offer photoprotection. More research on senescing leaves is obviously needed to clarify the possibly diverse functions of flavonols in deciduous plant species.

## MATERIALS AND METHODS

### Leaf material

At least 12 aphid-free and 5 aphid-infested leaves, including both green and yellow (senescing) leaves, were collected from the height of ∼1-2 m, from 10 mature silver birch (*B. pendula* Roth) and 4 mature downy birch (*B. pubescens* Ehrh.) trees, growing in city parks in Turku (Finland). Collection was conducted on September 20 - October 10, 2021, and September 21 - October 10, 2022. Aphids residing on the leaves (Fig. S17) were counted and removed, after which the leaves were brought to laboratory for further analyses. Between the collection and analyses (<3 h), leaves were kept in the dark, wrapped in a moist piece of paper.

### Pigment measurements

Leaf chlorophyll content was quantified with the optical SPAD method with MultispeQ v1 (PhotosynQ Inc., East Lansing, MI, USA). To validate and calibrate the SPAD measurements, leaf chlorophyll contents were measured, from a set of leaves, first with MultispeQ and then spectrophotometrically, according to Porra et al. (1989), after extraction of pigments in dimethylformamide as described by Mattila and Tyystjärvi (2023) (Fig. S18). The data were fitted to an empirical equation (Eqn 1; intercept=0; RMSE=2.79; Fig. S18) in Microsoft Excel. The Eqn 1 was then used to convert SPAD values to µg chlorophyll cm^−2^. In the case of the high light experiment (see below), chlorophyll contents were measured with another optical method, with Dualex Scientific™ (Force-A, Paris, France), and converted to µg chlorophylls cm^−2^ according to our earlier calibration curve (Eqn 2; Mattila et al., 2018).
(1)

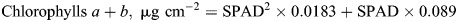

(2)

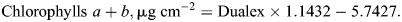
Total leaf flavonols were estimated optically with Dualex Scientific™; for a validation of the method, see Mattila et al. (2018). For measurements of individual flavonoid species and carotenoid to chlorophyll ratio, leaves were dried at 4°C in the dark, then ground, weighed and placed in methanol, as described in Mattila et al. (2018). Carotenoid to chlorophylls ratio was first measured spectrophotometrically according to Wellburn (1994) and Porra et al. (1989), respectively. Samples were then analysed with high-performance liquid chromatography (HPLC; Agilent 1100 Series, Agilent Technologies, Germany) according to Seal (2016), with modifications described in Mattila et al. (2018). Quantification of flavonoids was done with absorbance at 280 nm according to Seal (2016). A certain peak was classified as a flavonol if its absorption spectrum resembled those of known flavonol species (e.g. quercetin and rutin; Solovchenko, 2010).

### Photosynthetic parameters

F_V_/F_M_ (Eqn 3) was measured with FluorPen (Photon Systems Instruments, Drásov, Czech Republic), unless otherwise stated, from the collected leaves, after at least 30 min in the dark in the laboratory. After that, leaves were kept for a few minutes under low light [photosynthetic photon flux density (PPFD) of 10-20 µmol m^−2^ s^−1^] to activate photosynthesis, and then illuminated with white light of the PPFD 1000 µmol m^−2^ s^−1^ (from a low-voltage halogen lamp, equipped with a heat filter) for the measurements of other fluorescence parameters (Eqns 4-9), as well as for the quantification of active Photosystem I (PSI) centres from absorbance changes at 830 nm during a saturating flash, and leaf thickness, with MultispeQ (for more details, see Mattila and Tyystjärvi, 2023). The fluorescence parameters were calculated as follows:
(3)

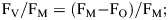

(4)

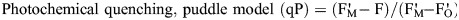

(5)

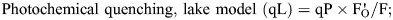

(6)

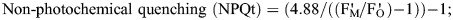

(7)

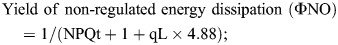

(8)

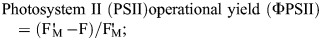


and
(9)

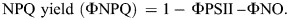
In Eqns 3-9, F_O_ and F_M_ are minimum (only a weak measuring beam on) and maximum (during a saturating pulse) fluorescence yields, respectively, measured from a dark-acclimated sample, and F_O_’ and F_M_’ are minimum (under far-red light) and maximum (during a saturating pulse) fluorescence yields measured from a light-acclimated sample. F is fluorescence yield under illumination. Due to low signal to noise ratio, measurements of qL from leaves with low chlorophyll content (<5 µg cm^−2^) have been removed prior any analyses.

### High light treatment

Aphid-free silver birch leaves were collected on September 29 - October 6, 2022, from three to four trees and flavonols and chlorophyll contents were measured with Dualex Scientific™; chlorophyll contents were converted to µg cm^−2^ according to Eqn 2 (see Mattila et al., 2018). From each tree, the green and senescing leaf with the highest and the lowest flavonol content among the green and senescing leaves of that tree were selected to obtain groups of green and senescing leaves with high and low flavonol content. The experiment was repeated on four different dates. Leaves were illuminated for 1.5 h with high light (PPFD 2000 µmol m^−2^ s^−1^) from a sunlight simulator (SL Holland), on top of a wet paper placed on a temperature-controlled metal block (set to 20°C) and let to recover for 2 h, on top of a wet paper at room temperature at low light (PPFD 12 µmol m^−2^ s^−1^). Before and after the high light treatment and after the recovery, leaves were dark-acclimated for 30 min and the F_V_/F_M_ values were measured with Dual-Klas-NIR fluorometer (Walz, Germany), as described in Mattila et al. (2021).

### Statistics

The dispersion of the aphid data (the number of aphids on a leaf) was tested with the dispersiontest function (AER package; Kleiber and Zeileis, 2008) of R (R Core Team, 2021) and a linear model assuming a negative binomial distribution, constructed with the MASS package (Venables and Ripley, 2002) was used for the analysis. Linear mixed models, constructed with the lme4 package (Bates et al., 2015) were used for the analysis of flavonol content, Φ(NPQ), relative amount of active PSI centres and leaf thickness. For F_V_/F_M_, a beta regression model, constructed with the betareg R package (Cribari-Neto and Zeileis, 2010) was used. For the used variables, complete results and diagnostic figures, see Figs S1-S16, Table 3 and Tables S1-S8.

Statistically significant differences for the photoinhibition data were tested by calculating the Mann–Whitney *U-*test with Microsoft Excel using the Real Statistics Resource Pack (Zaiontz, 2020). Prior the analyses, few leaves with (too) high or low chlorophyll content were removed, to obtain comparable (in terms of chlorophyll contents) groups to reliably estimate the effects of flavonols.

Asterisks indicate *P*<0.05 (*), *P*<0.01 (**) or *P*<0.001 (***).

## Supplementary Material

10.1242/biolopen.060325_sup1 Supplementary information
