# Supplementary material for: Flavonols do not affect aphid load in green or senescing birch leaves but coincide with a decrease in Photosystem II functionality
Source: Biol Open. 2024 Jul 2;13(7):bio060325. doi: 10.1242/bio.060325 (PMC11261631; doi:10.1242/bio.060325)
Supplement: Supplementary information [file biolopen-13-060325-s1.pdf]

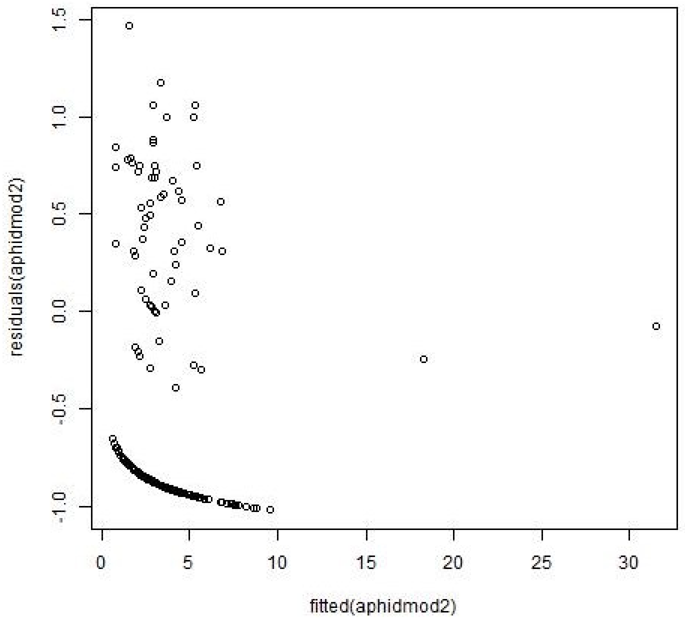

Fig. S1. Residuals of the model for aphid number on a leaf versus fitted values of the model.

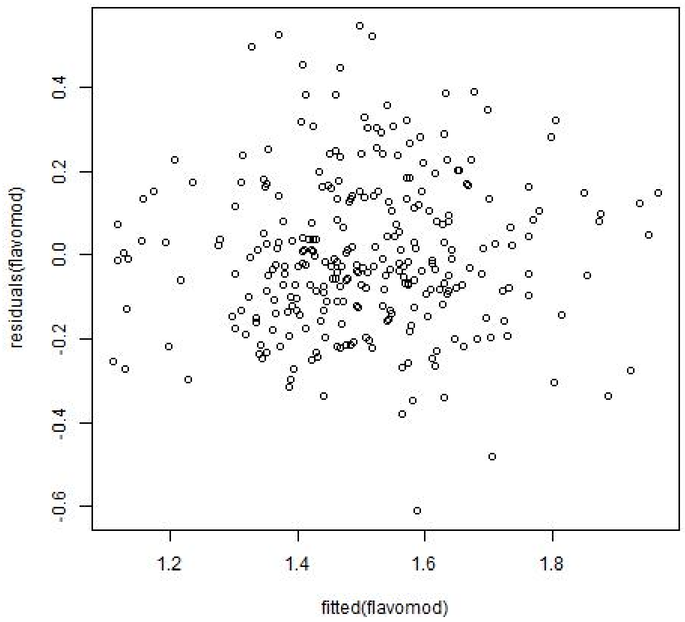

Fig. S2. Residuals vs fitted values of the flavonol model.

Biology Open • Supplementary information

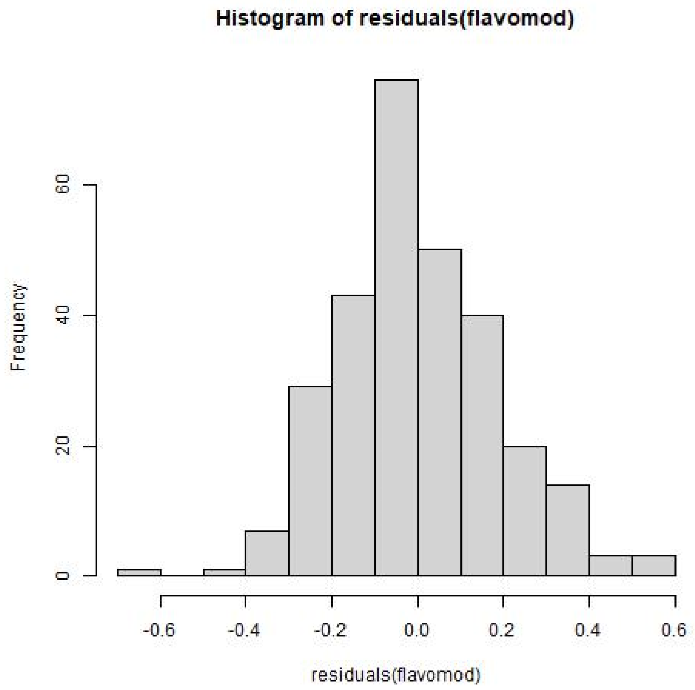

Fig. S3. Residuals of the flavonol model.

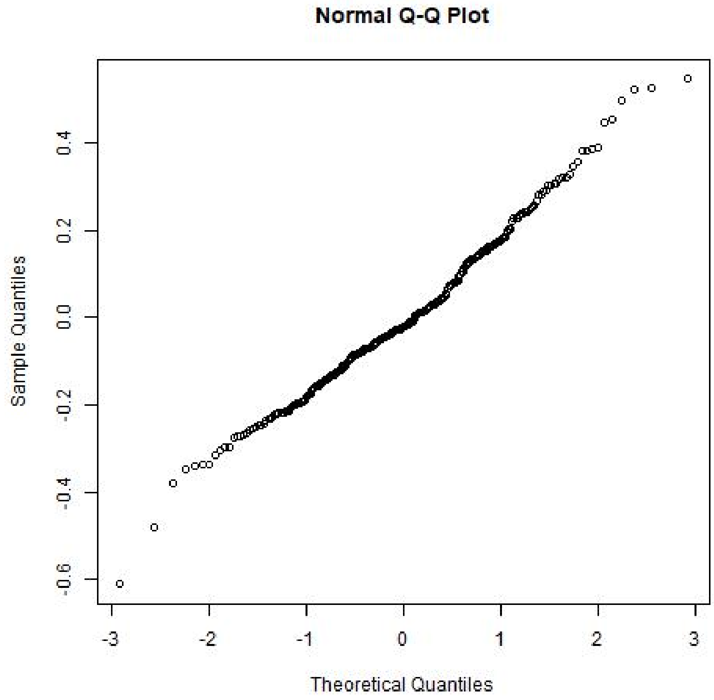

Fig. S4. Quantiles of the flavonol model as a function of theoretical quantiles of the same distribution.

Biology Open • Supplementary information

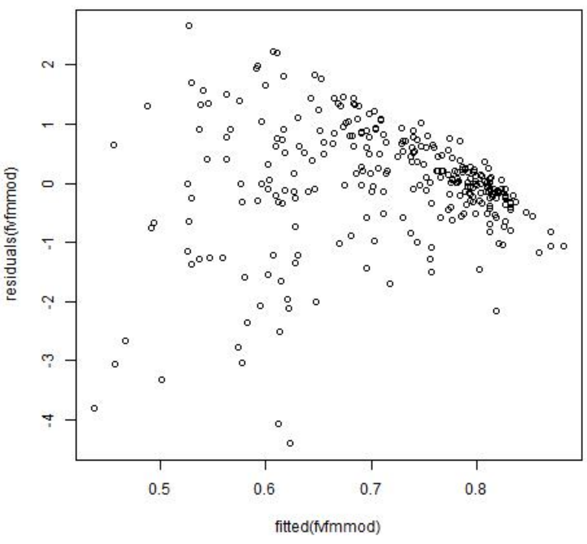

Fig. S5. Residuals of the F_V/F_M model as a function of fitted values.

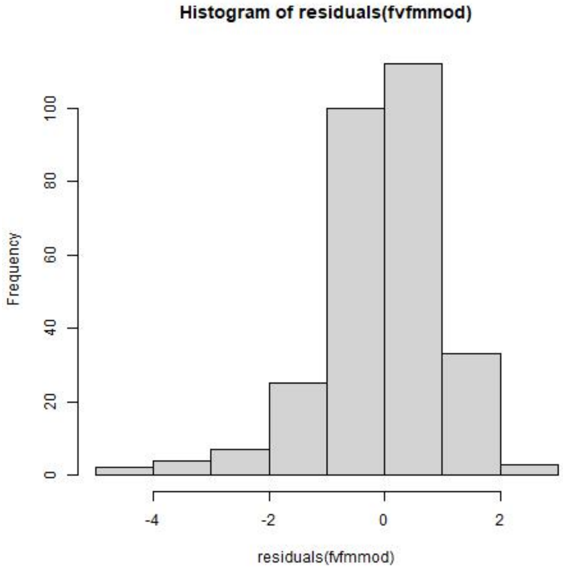

Fig. S6. Residuals of the model for F_V/F_M.

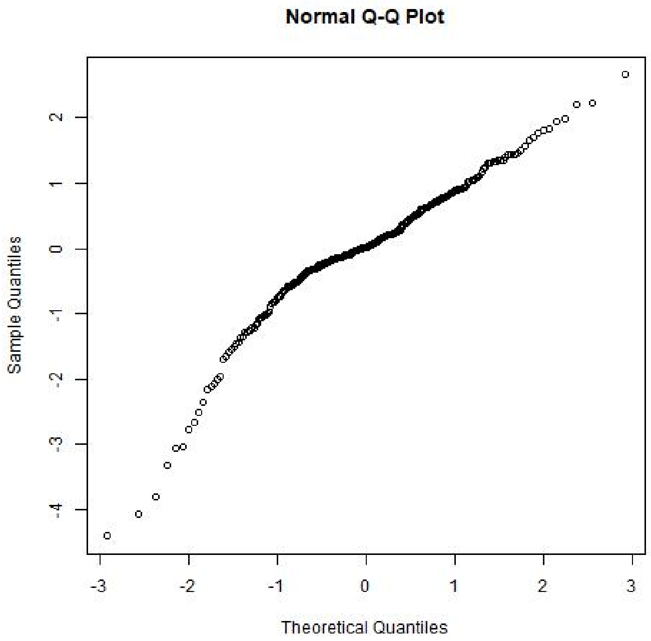

Fig. S7. Quantiles of the residuals of model for F_V/F_M as a function of normally theoretical quantiles.

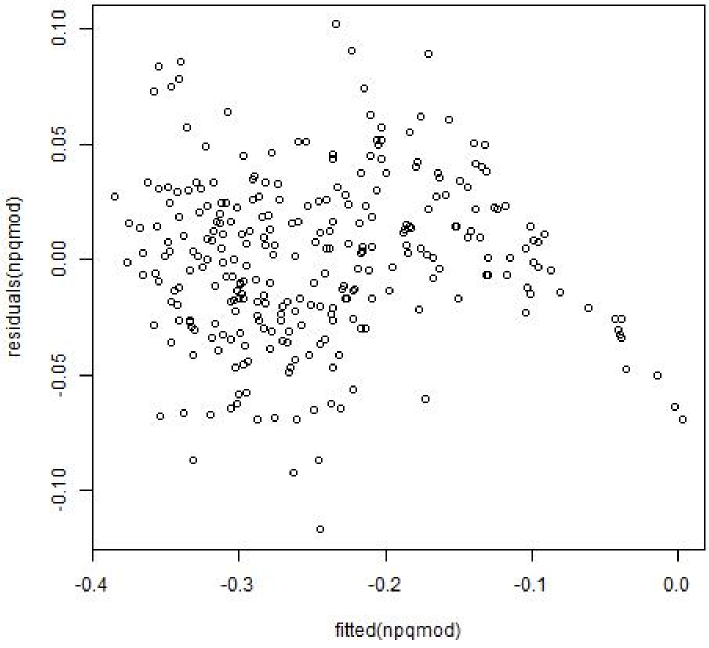

Fig. S8. Residuals of the model for NPQ as a function of fitted values.

Biology Open • Supplementary information

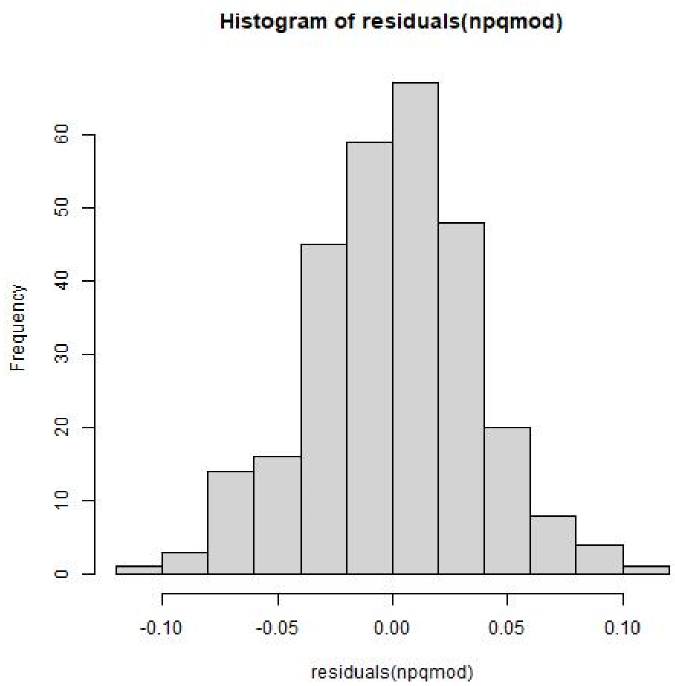

Fig. S9. Residuals of the model for NPQ.

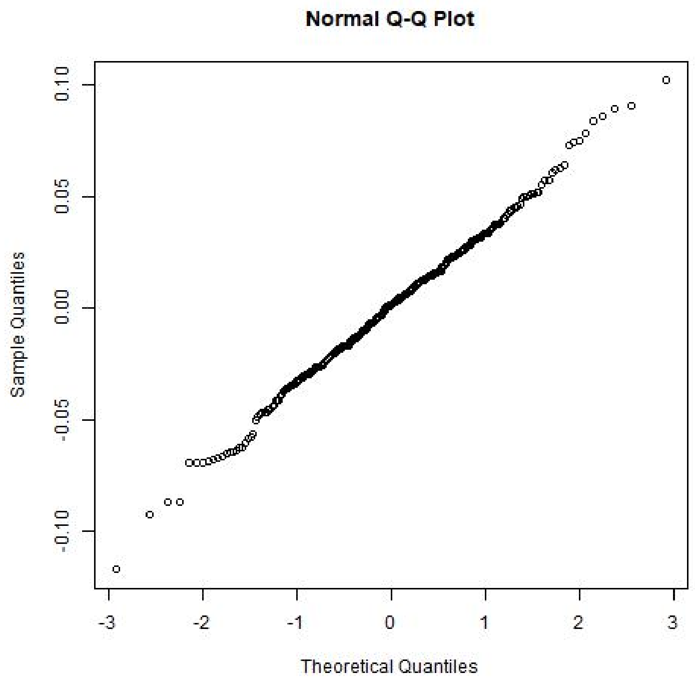

Fig. S10. Quantiles of the model for NPQ as a function of theoretical quantiles of the same distribution.

Biology Open • Supplementary information

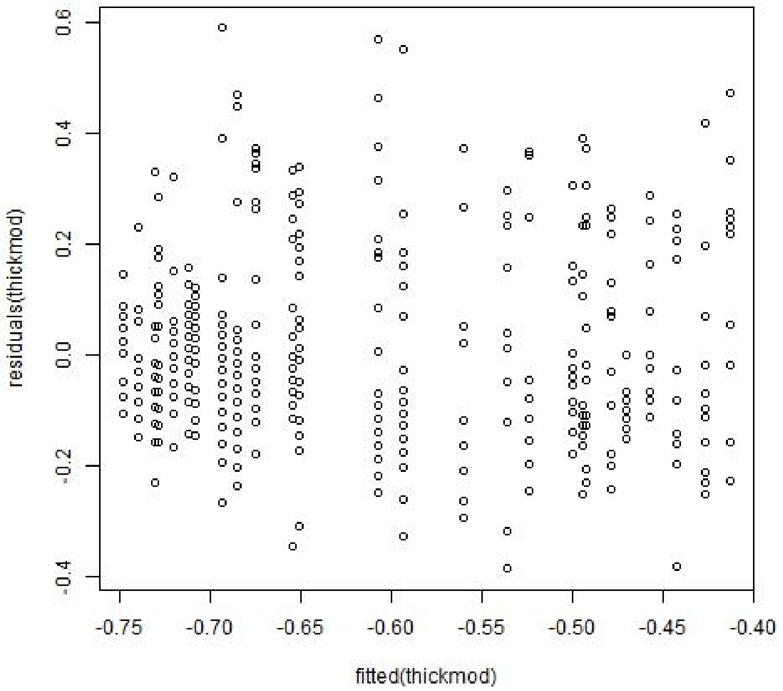

Fig. S11. Residuals of the model for leaf thickness as a function of the fitted values.

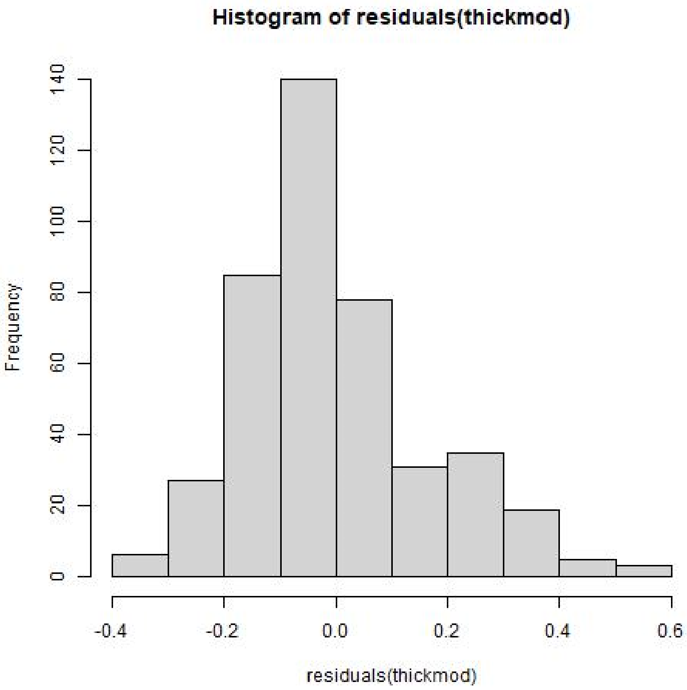

Fig. S12. Residuals of the model for leaf thickness.

Biology Open • Supplementary information

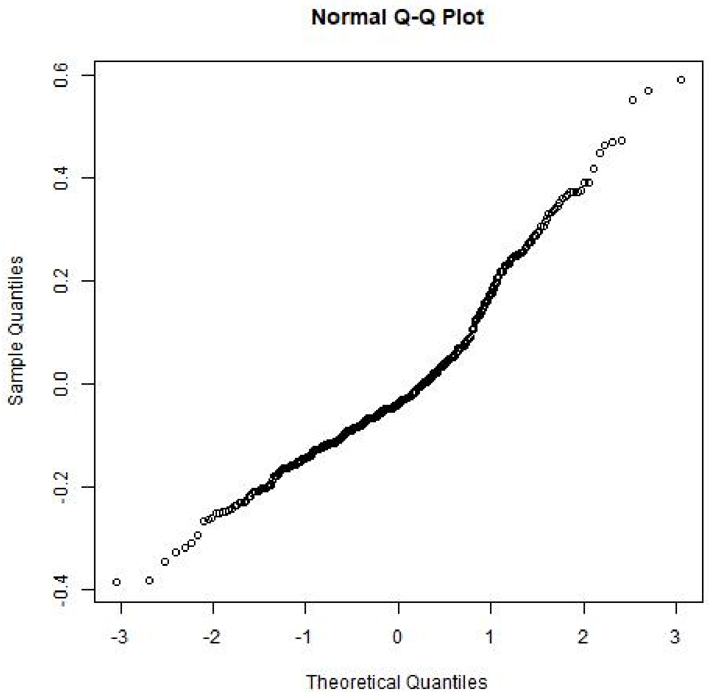

Fig. S13. Quantiles of the model of leaf thickness as a function of theoretical quantiles.

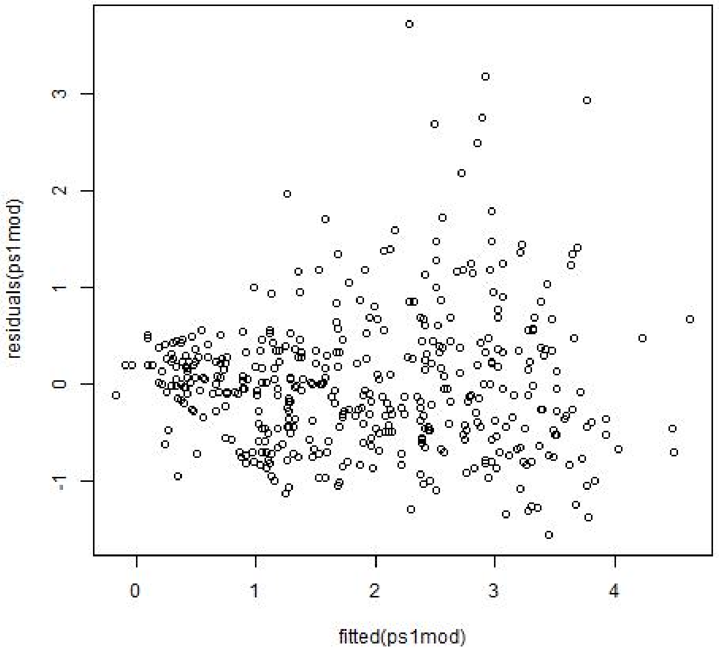

Fig. S14. Residuals of the model for PSI centres as a function of the fitted values.

Biology Open • Supplementary information

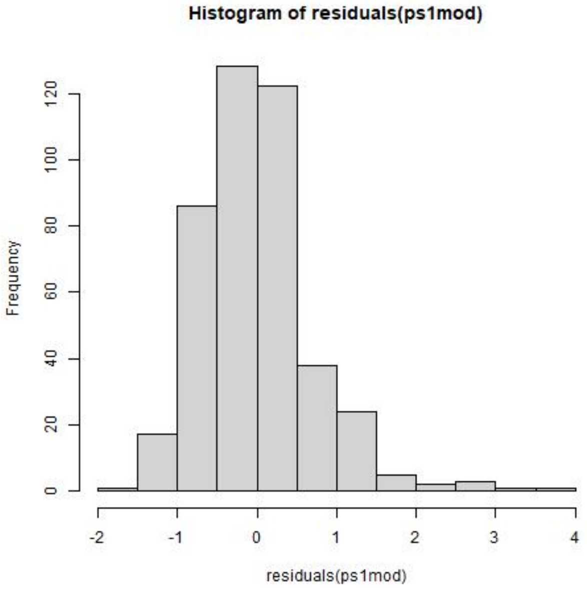

Fig. S15. Residuals of the model for PSI centres.

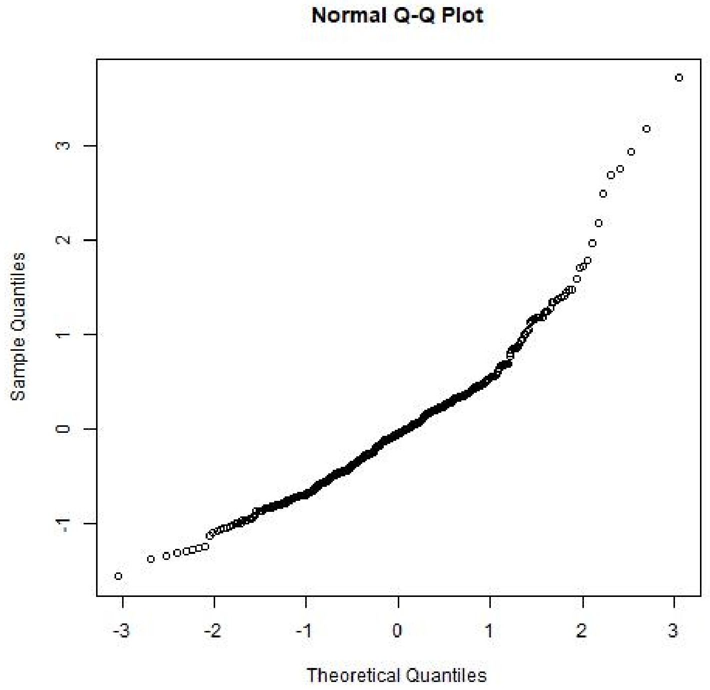

Fig. S16. Quantiles of the model for PSI centres as a function of theoretical quantiles.

Biology Open • Supplementary information

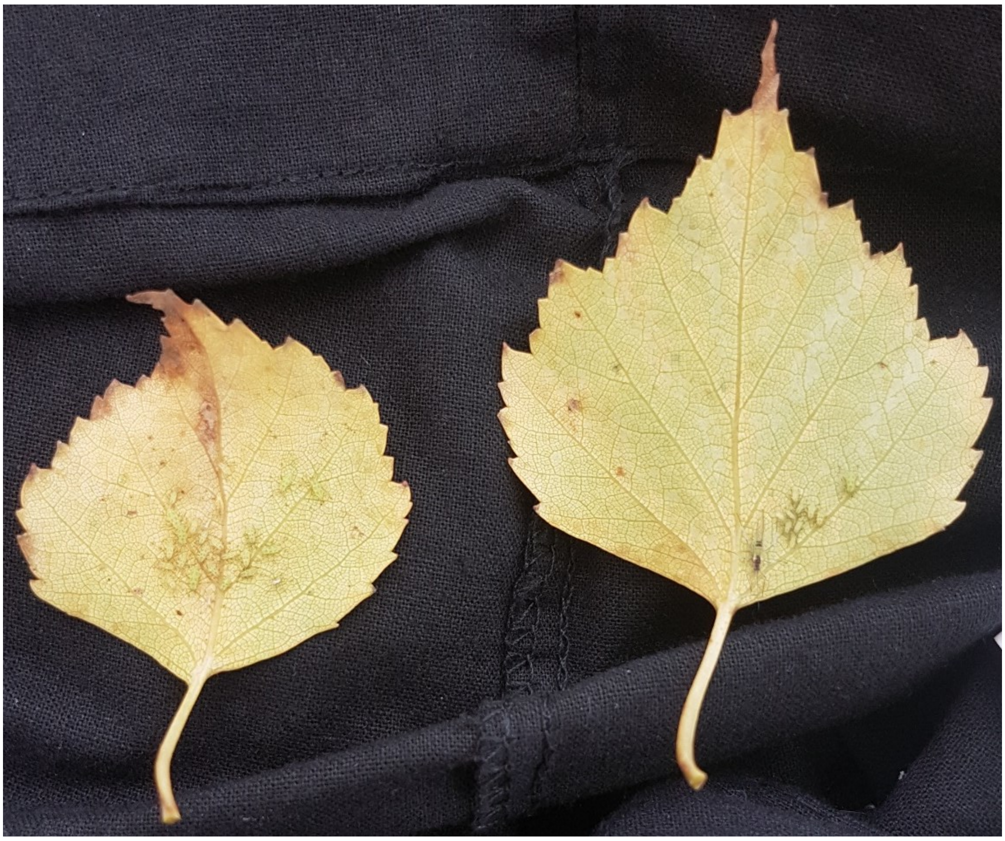

Fig. S17. Aphids residing on the abaxial (bottom) surfaces of senescing silver birch leaves. ~20 nymphs can be seen on the leaf to the left, and ~10 nymphs and a winged adult can be seen on the leaf to the right.

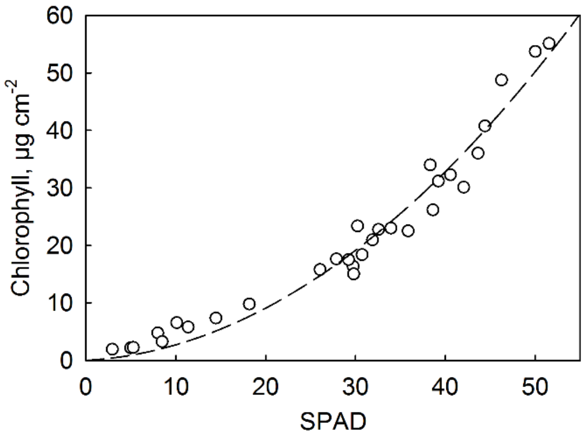

Fig. S18. Calibration of the optical chlorophyll measurements (SPAD). After the optical measurements with MultispeQ were conducted on birch leaves containing different amounts of chlorophyll, pigments were extracted in dimethylformamide and quantified spectrophotometrically. Symbols represent individual measurements from single leaves, collected from three trees, and the dashed line shows the best fit to the following polynomial equation: chlorophylls *a* + *b*, µg cm^{-2} = $SPAD^2$ x 0.0183 + SPAD x 0.089.

Table S1. Overdispersion test of the number of aphids per birch leaf. The leaves were collected from Turku, Finland during the autumns of 2021 and 2022 and aphids were counted on each leaf. The overdispersion test was done with the "dispersiontest" R function.

z	p	Dispersion
4.5914	2.202E-6	15.2884

Table S2. Effects of the measured parameters on the number of aphids per birch leaf. The leaves were collected from Turku, Finland during the autumns of 2021 and 2022 and aphids were counted on each leaf. The model assumed a negative binomial distribution for the response variable, using the R call aphidmod1 = glm.nb(APHIDS ~ FLAV + CHL + FVFM + QL+ THICKNESS + PS1ACT + RAUDUS + JULIAN, data=aphids, init.theta=1.05, link=log), where FLAV is the amount of flavonols, CHL is the chlorophyll content, QL is the qL fluorescence parameter, THICKNESS is leaf thickness, PS1ACT is the number of active PSI centres, RAUDUS is the birch species (1=*B. pendula*, 0=*B. pubescens*) and JULIAN is the day of the year. The deviance residuals were Min: -1.0172, 1Q: -0.9003, Median: -0.8093, 3Q: -0.1308, and Max: 1.4635. Null deviance was 139.57 on 211 degrees of freedom and residual deviance 132.40 on 203 degrees of freedom. Akaike Information Criterion: 714.6; Theta: 0.1171, Std. error: 0.0184; 2 x log-likelihood: -694.5980. The dispersion parameter for negative binomial (0.1171) family was taken to be 1.

	Estimated coefficient	Std. error	Z value	Pr(>|z|)
(Intercept)	-17.5425	10.94004	-1.604	0.109
FLAV	0.79852	1.08403	0.737	0.461
CHL	-0.03212	0.03044	-1.055	0.291
FVFM	5.84583	4.98873	1.172	0.241
QL	-7.82318	19.24968	-0.406	0.684
THICKNESS	2.58294	2.18492	1.182	0.237
PS1ACT	-0.06498	0.23291	-0.279	0.78
RAUDUS	-0.25349	0.5099	-0.497	0.619
JULIAN	0.0506	0.0354	1.429	0.153

Table S3. Effects of the measured parameters on the presence of aphids on a birch leaf. The leaves were collected from Turku, Finland during the autumns of 2021 and 2022 and aphids were counted on each leaf. The presence of aphids was coded as 1 and the absence as 0. The model assumed a Poisson distribution for the response variable, using the R function aphidmod2 = glm(formula = PRESENCE ~ FLAV + CHL + FVFM + THICKNESS + PS1ACT + RAUDUS + QL, family = poisson(link = "log"), data = aphids), where FLAV is the amount of flavonols, CHL is the chlorophyll content, QL is the qL fluorescence parameter, THICKNESS is leaf thickness, PS1ACT is the number of active PSI centres, RAUDUS is the birch species (1=*B. pendula*, 0=*B. pubescens*). The deviance residuals were Min: -1.3187, 1Q: -0.7800, Median: -0.6912, 3Q: 0.7995, and Max: 1.4575. Null deviance: 152.89 on 211 degrees of freedom, residual deviance: 147.48 on 204 degrees of freedom; 217 observations deleted due to missingness; Akaike information criterion: 289.48.

	Estimated coefficient	Std. error	Z value	Pr(>|z|)
(Intercept)	-2.05307	2.65936	-0.772	0.44
FLAV	0.09523	0.66723	0.143	0.887
CHL	-0.02276	0.0187	-1.217	0.224
FVFM	0.62859	2.98956	0.21	0.833
THICKNESS	1.67156	1.02136	1.637	0.102
PS1ACT	0.14062	0.10853	1.296	0.195
RAUDUS	0.05805	0.3072	0.189	0.85
QL	0.99461	11.57399	0.086	0.932

Biology Open • Supplementary information

Table S4. Effects of the other measured parameters on F_V/F_M of a birch leaf. The leaves were collected from Turku, Finland during the autumns of 2021 and 2022 and analysed for flavonol content (FLAV), chlorophyll content (CHL), number of active PSI centres (PS1ACT), leaf thickness (THICKNESS) and number of aphids per leaf (APHIDS). A linear model assuming a beta distribution for the response variable was built, using the R call FVFMModel = betareg(formula = FVFM ~ FLAV + CHL + FLAV * CHL + PS1ACT + THICKNESS + APHIDS, data = aphids). A maximum likelihood estimator was used, and the log likelihood was 289.4 on 8 degrees of freedom (pseudo $r^2 = 0.5268$). Standardized weighted residuals were Min: -4.4024; 1Q: -0.3576; Median: 0.0154; 3Q: 0.6215, and Max: 2.6567.

	Estimated coefficient	Std. error	Z value	Pr(>|z|)	Signif.
(Intercept)	2.615894	0.290629	9.001	<2E-16	***
FLAV	-1.37782	0.173688	-7.933	2.14E-15	***
CHL	-0.04075	0.011106	-3.669	0.000243	***
PS1ACT	0.011266	0.03367	0.335	0.737927	
THICKNESS	-0.24712	0.230363	-1.073	0.283391	
APHIDS	-0.00422	0.00348	-1.212	0.225434	
FLAV:CHL	0.044418	0.007567	5.87	4.37E-09	***

Table S5. Effects of the other measured parameters on Φ(NPQ) of a birch leaf. The leaves were collected from Turku, Finland during the autumns of 2021 and 2022 and analysed for flavonol content (FLAV), chlorophyll content (CHL), number of active PSI centres (PS1ACT), leaf thickness (THICKNESS) and number of aphids per leaf (APHIDS). The tree individual (14 trees, 286 observations) was used as a grouping variable. The response variable was log-transformed. A linear mixed model assuming a Gaussian distribution for the response variable was built, using the R call npqmod = lmer(LOGYNPQ ~ FLAV + CHL + FVFM + PS1ACT + THICKNESS + RAUDUS + JULIAN + (1|TREE), data=aphids), where JULIAN is the day of the year. The REML criterion at convergence was -997.4 and the scaled residuals were Min: -3.2136, 1Q: -0.6249, Median: 0.0440, 3Q: 0.6385, and Max: 2.7953. Log likelihood tests, applied using the "anova" function of R by omitting the parameter under study from a null model and then comparing the full and the null models, were used to test the significance of each fixed effect. AIC, BIC, log likelihood and Chisq for the full model were -1052.2, -1015.6, 536.08 and -1072.2, respectively. The maximum likelihood criterion, instead of REML, was used for fitting the models used for the anova. A significance test could not be run (nd) for FLAV because the datasets of the full and null model were of different sizes.

**Groups**							
	**Name**	**Variance**	**Std. dev.**				
TREE	(Intercept)	0.000199	0.01409				
Residual		0.001326	0.03642				
**Fixed effects**							
	**Estimated coefficient**	**Std. error**	**t value**	**Chisq**	**Pr (>Chisq)**	**Signif.**	
(Intercept)	-0.02756	0.177287	-0.155	2.45	0.118		
FLAV	-0.00499	0.011325	-0.441	nd	nd	nd	
CHL	-0.00241	0.000263	-9.15	75.5	<2E-16	***	
FVFM	-0.30662	0.021337	-14.37	159.2	<2E-16	***	
PS1ACT	-0.00649	0.002948	-2.202	4.92	0.027	*	
THICK-NESS	-0.03414	0.019991	-1.708	3.22	0.072		
RAUDUS	0.005946	0.009814	0.606	0.482	0.487		
JULIAN	0.000265	0.000648	0.41	0.199	0.656		

Biology Open • Supplementary information

**Characteristics of null models**							
	**AIC**	**BIC**	**Log likely-hood**	**Devianc e**			
-CHL	-978.63	-945.73	498.31	-996.63			
-FVFM	-894.92	-862.01	456.46	-912.92			
-PS1ACT	-1049.2	-1016.3	533.62	-1067.2			
-THICK-NESS	-1050.9	-1018.0	534.46	-1068.9			
-RAUDUS	-1053.7	-1020.8	535.84	-1071.7			
-JULIAN	-1054.0	-1021.0	535.98	-1072.0			
**Correlation of fixed effects**							
	(Intr)	FLAV	CHL	FVFM	PS1ACT	THICK-NESS	RAUDUS
FLAV	-0.002						
CHL	-0.026	0.088					
FVFM	-0.268	0.148	-0.316				
PS1ACT	0.153	0.015	-0.693	-0.163			
THICK-NESS	-0.162	0.011	-0.012	0.131	0.016		
RAUDUS	0.261	-0.188	0.096	-0.058	-0.102	-0.014	
JULIAN	-0.99	-0.104	0.034	0.182	-0.149	0.126	-0.275

Biology Open • Supplementary information

Table S6. Effects of chlorophyll content (CHL), number of days from the beginning of the year (JULIAN) and the number of aphids on the leaf (APHIDS) on the number of active PSI centres of birch leaves. The leaves were collected from Turku, Finland during the autumns of 2021 and 2022. The tree individual (TREE, 14 groups) and YEAR (2 groups) were used as grouping parameters in a linear mixed model, obtained with the R call ps1mod = lmer(PS1ACT ~ CHL + JULIAN + APHIDS + (1 | TREE) + (1 | YEAR)). The REML criterion for convergence was 1012.6 and the scaled residuals were Min: -2.1374, 1Q: -0.6521, Median: -0.0793, 3Q: 0.4540, and Max: 5.0594. The significance of each estimated coefficient was tested with a maximum likelihood test with the "anova" function of R, by comparing a null model from which the effect under study was omitted and the full model. AIC, BIC, log likelihood and deviance of the full model were 999.7, 1028.1, -492.85 and 985.7, respectively.

**Groups**						
	**Name**	**Variance**	**Std. dev.**			
TREE	(Intercept)	0.1943	0.4408			
YEAR	(Intercept)	0.3272	0.5720			
Residual		0.5388	0.7340			
**Fixed effects**						
	**Estimated coefficient**	**Std. error**	**t value**	**Chisq**	**Pr(>Chisq)**	**Signif.**
(Intercept)	0.353235	2.147589	0.164			
CHL	0.073282	0.002637	27.789	436.49	<2.2e-16	***
JULIAN	0.001688	0.007626	0.221	0.0797	0.7777	
APHIDS	-0.007399	0.005296	-1.397	1.9751	0.1599	
**Characteristics of the null models**						
	**AIC**	**BIC**	**Log likelihood**	**Deviance**		
-CHL	1434.2	1458.5	-711.10	1422.2		
-JULIAN	997.78	1022.1	-492.89	985.78		
-APHIDS	999.68	1024.0	-493.84	987.68		
**Correlation of fixed effects**						
	(Intr)	CHL	JULIAN			
CHL	-0.158					
JULIAN	-0.980	0.139				
APHIDS	-0.001	0.111	-0.009			

Biology Open • Supplementary information

Table S7. Effects of the birch species and day of the year on leaf thickness. The birch species was coded by the RAUDUS parameter as 1=*B. pendula*, 0=*B. pubescens*, and the day of the year was log-transformed (LOGJULIAN). The response variable was also log-transformed. Tree individual (TREE, 14 groups), and YEAR (2 groups); 429 observations were used as grouping parameters in a linear mixed model assuming a Gaussian distribution of the response variable, using the R call thickmod = lmer(LOGTHICKNESS ~ RAUDUS + LOGJULIAN + (1|YEAR) + (1|TREE), data=aphids). The scaled residuals were Min: -2.2861; 1Q: -0.6578; Median: -0.2223; 3Q: 0.4203; Max: 3.5128. The REML criterion at convergence was -284.2. The significance of each fixed effect was tested by comparing, with the "anova" function of R, a null model with the parameter under study omitted to the full model. The REML criterion was relaxed for the tests. For the full model, AIC, BIC, Log likelihood and deviance were -278.97, -254.60, 145.49 and -290.97, respectively.

**Groups**						
	**Name**	**Variance**	**Std. dev.**			
TREE	(Intercept)	0.002218	0.0471			
YEAR	(Intercept)	0.015178	0.1232			
Residual		0.02832	0.1683			
**Fixed effects**						
	**Estimated coefficient**	**Std. error**	**t value**	**Chisq**	**Pr(>Chisq)**	**Signif.**
(Intercept)	4.97111	2.267434	2.192			
RAUDUS	0.003325	0.033045	0.101	0.0098	0.921	
LOGJULIAN	-2.29023	0.931076	-2.46	5.7106	0.01686	*
**Characteristics of the null models**						
	**AIC**	**BIC**	**Log likelihood**	**Deviance**		
-RAUDUS	-280.96	-260.66	145.48	-290.96		
-LOGJULIAN	-275.26	-254.96	142.63	-285.26		
**Correlation of fixed effects**						
	(Intr)	RAUDUS				
RAUDUS	0.198					
LOGJULIAN	-0.999	-0.207				

Biology Open • Supplementary information

Table S8. Effects of other measured parameters on flavonol content of a birch leaf. The leaves were collected from Turku, Finland during the autumn of 2022 and analysed for flavonol content (FLAV), chlorophyll content (CHL), F_V/F_M (FVFM), number of active PSI centres (PS1ACT), leaf thickness (THICKNESS). Two birch species were used, coded with the RAUDUS parameters as *B. pendula*=1, *B. pubescens*=0. A linear mixed model with an assumed Gaussian base distribution for the response variable was built, with the tree individual (TREE; 14 trees among 287 observations) as a grouping variable. The R call was flavomod = lmer(FLAV ~ CHL + FVFM + THICKNESS + PS1ACT + RAUDUS + (1|TREE), data=aphids). The REML crite criterion at convergence was -85.1. Scaled residuals: Min: -3.2258; 1Q: -0.6541; Median: -0.1109; 3Q: 0.6685; Max: 2.8809. The significance of each estimated coefficient was tested with likelihood ratio tests done by comparing with the "anova" function of R, the full model to a model from which the effect under study was omitted and the ML criterion was used for fitting. For this test, AIC, BIC, log likelihood and deviance for the full model were -102.40, -73.12, 59.2 and -118.4, respectively. The df for the tests was 1, with 8 parameters in the full model and 7 in the null model.

**Groups**						
	**Name**	**Variance**	**Std. dev.**			
TREE	(Intercept)	0.01171	0.1082			
Residual		0.03586	0.1894			
**Fixed effects**						
	**Estimated coefficient**	**Std. error**	**t value**	**Chisq**	**Pr (>Chisq)**	**Signif.**
(Intercept)	1.642649	0.091254	18.001			
CHL	-0.00214	0.001378	-1.551	2.45	0.118	
FVFM	-0.29222	0.10947	-2.669	7.33	0.007	**
THICKNESS	-0.03583	0.104044	-0.344	0.131	0.718	
PS1ACT	-0.00076	0.01545	-0.049	0.001	0.972	
RAUDUS	0.190588	0.065261	2.92	7.622	0.006	**
**Characteristics of null models**						
	**AIC**	**BIC**	**Log likelihood**	**Deviance**		
-CHL	-101.95	-76.331	57.974	-115.95		
-FVFM	-97.071	-71.455	55.536	-111.07		
-THICKNESS	-104.27	-78.651	59.134	-118.27		
-PS1ACT	-104.4	-78.780	59.198	-118.4		
-RAUDUS	-96.776	-71.160	55.388	-110.78		
**Correlation of fixed effects**						
	(Intercept)	CHL	FVFM	THICK-NESS	PS1ACT	RAUDUS
CHL	0.17					
FVFM	-0.751	-0.338				
THICKNESS	-0.371	-0.01	0.122			
PS1ACT	0.081	-0.701	-0.159	0.018		
RAUDUS	-0.481	0.101	0.025	0.023	-0.117	

Biology Open • Supplementary information
